# Selection of Solar Powered Unmanned Aerial Vehicles for a Long Range Data Acquisition Chain

**DOI:** 10.3390/s21082772

**Published:** 2021-04-14

**Authors:** Wiktor Woźniak, Mieczysław Jessa

**Affiliations:** Faculty of Computing and Telecommunications, Poznań University of Technology, 60-965 Poznań, Poland; wiktor.ma.wozniak@doctorate.put.poznan.pl

**Keywords:** unmanned aerial vehicles, data acquisition, sensors, solar energy, photovoltaic cell, renewable energy, energy harvesting, wireless sensor network, distributed measurement network, radio relay network, network chain, routing protocols, environment monitoring, agriculture

## Abstract

The paper presents a long range data acquisition chain operating in areas without access to the electricity grid or communication infrastructure built with unmanned aerial vehicles (UAVs). It is assumed that the length of the network chain significantly exceeds the flight range of a single drone. To build such a network three basic problems have to be solved. The first is energy harvesting for battery charging. The second concerns the choice of drone models that can cover a given distance in the shortest time. The third problem is the reduction of the flight range of drones as a function of payload mass. The evaluation of the proposed method is based on the results of simulations and cost analysis of 54 drones and 25 solar cells. The analysis ends with a proposition of seven steps that can help to choose the most suitable drone model for a given task.

## 1. Introduction

As a result of rapid technological progress, unmanned aerial vehicles (UAVs) have become more and more popular. UAVs have a variety of applications, ranging from military to commercial and civilian ones. They have multiple real-life applications like aerial photography, cartography and geodesy, precision farming, life rescue missions, infrastructure inspection and environmental protection [1]. UAVs can be piloted remotely or fly autonomously along a planned path. They are used by the military to observe enemy-controlled areas and, in civil applications, to transmit radio signals from difficult to access territories, where it is uneconomic to build a network of terrestrial transmitters, for example television, telecommunications and relay stations [2,3].

The literature describes a drone as an unmanned aerial vehicle which does not require a pilot presence on board. This kind of vehicle is not allowed to transport passengers. It is piloted remotely or performs autonomous flights. Both taking-off and landing (or recovery) phases of the drone take place with automatic systems or are controlled by an external operator [4].

Modern classification of UAVs consists of the following categories: (a) airplanes; (b) helicopters; (c) multirotors; (d) airships and (e) other unmanned aerial vehicles. Each of these categories can be propelled with fossil fuels or electricity. Unlike fossil fuel powered models, electric energy powered ones do not emit any pollutants into the environment. A hybrid drone is also described in the literature where both a fuel-based engine and an electric motor are used to propel a propeller [5,6]. By generalizing the division of aerial vehicles, a distinction between winged and rotor-equipped ones can be made. Airplanes and gliders represent examples of winged aerial vehicles. This type is characterized by the need of using a launcher. Rotor vehicles are holonomic—they are able to start and land vertically without any launcher [7].

Following the idea of electrically-powered UAV usage, there is a need to provide the necessary energy. A typical UAV movement scenario assumes that the drone flies from a ground site to the desired position, serves selected areas, returns to the ground site, and recharges its battery at the ground site. This forces one to schedule UAV missions in a way to preserve their battery power level as much as possible [3]. The most common and the simplest solution is battery replacement by an operator and then charging them externally in a docking station or using a built-in drone charger with a connected power cable. In the case of the winged vehicles it can be easily solved by covering the wings with solar panels [8]. As an example, Google’s project called SkyBender providing drone-powered small cellular networks can be mentioned [9]. During the day, the energy is collected in accumulators and consumed for the current flight. During the night, the motors and network devices are powered by batteries. As a result, there is a winged UAV that flies and can provide Internet access for the whole day. Another example of a winged UAV is described in [10], where the authors proposed the usage of flexible solar cells embedded on the UAV without impacting its aerodynamics. Most of the battery powered UAVs use Li-polymer type batteries which were evaluated in [11].

Sending electric drones into an area without a power grid requires the energy harvesting problem to be solved. There is no option for an operator to replace the battery. In-flight wireless charging using two loop antennas, the first one installed on the ground and the second one on the drone’s body, is limited to the range of wireless power signal transmission [12]. When the drone is located within the ground antenna’s range, it gathers energy and recharges its batteries. A similar solution was used in [13], where the authors proposed UAVs to be used as base stations to cover certain areas of interest where the signal from a traditional base station cannot reach. The trade-off between the size of the service area and in-flight charging power was discussed. A stationary drone box charging station with solar power harvesting can also be considered [14]. A disadvantage of this method is the need to transport system elements to the desired operation site to keep the drone within the range of a wireless power station. A more appropriate solution is an independent charging system merged with the drone. With the development of new technologies, RF energy harvesting from the existing radio environment [15] seems to be a prospective source of energy for drones with small capacity batteries. Traditional energy harvesting technologies were mostly concentrated on solar energy or wind energy. Examples of solar powered UAVs used for environmental monitoring in Thailand or sub-Saharan Africa were described in [16,17], respectively. In [18] a charging system consisting of a PV panel and a fuel cell was proposed. The PV panel feeds extra energy back to the battery with higher priority and to an electrolyzer to produce hydrogen for any night operations.

In terms of wireless networks, a special example of UAV usage for collecting data from water meters using IEEE 802.15.4 ZigBee combined with a Raspberry Pi microcomputer was described in [19]. Cellular networks can also be UAV-aided. Small cells mounted on top of an UAV are described in [20]. A few disaster management applications were proposed in [21]. Drones are used for monitoring, forecasting and early warning activities. When a disaster happens, drones can provide a bridge between different information technologies or can be used to build a standalone communication system.

The authors of this paper, in response to the statement presented by Zhou et al. [22]: “*industrial unmanned aerial vehicles (UAVs), which enable autonomous inspection and measurement of anything anytime anywhere*”, propose to use a chain of drones to collect data for non-urbanized areas without electricity access, inaccessible to a single drone because of its limited range or unacceptable costs. An unquestionable advantage of drones is the possibility to reach inaccessible places on Earth in a relatively short time. Using sensors, they can monitor the environment at such places with simultaneous transmission of data to a base station. The position of drones can be changed on demand with the use of suitable commands and protocols. Drones gather and transmit data to the base station (BS) in real time. The length of the whole network chain significantly exceeds the flight range of a single drone. The system is designed to operate in poorly accessible areas like glaciers, deserts, high mountains, conflict zones, etc. It is theoretically possible for people to get there, but it takes a lot of time and the journey may be dangerous to their health or life. To the best of the authors’ knowledge, this problem has not yet been considered in the literature.

The proposed system can be applied for remote monitoring of environmental parameters, for example, pollutants, temperature and humidity. The possibility of usage in agriculture is also noted. Relaying data from remote weather or water stations as a way of establishing a relatively low-cost network can also be considered. Using a network chain carried by drones it is also possible to bypass broken network links. Drones are able to set up a new network for disaster management and provide connectivity throughout the affected area. The proposal is particularly useful in military applications where energy is not available and the area may be extremely dangerous.

The paper is organized as follows: Section 2 describes the concept, the system model, the operation algorithm, network architectures and possible data routing protocols. Section 3 introduces the topic of UAVs, where the classification and selected examples are presented, discusses the energy harvesting problem for drone powering and battery charging, gives the simulation results of the set of panels applied to the set of drones, and presents the method of computing the total time of building a network chain with drones supplied with a photovoltaic panel. The solution to the problem of the drone’s flight range reduction caused by the payload mass is presented in Section 4. The paper ends in Section 5 with resulting conclusions.

## 2. Concept and Method

The aim of the described system is to wirelessly transmit small portions of data for the longest possible distance, denoted as *D*, under some special conditions. Because we consider an area without electricity and possible gaps in communication infrastructure, the system has to be self-sustaining in terms of energy management and networking. The second condition is the ability to reach places that are difficult or/and dangerous to access for humans. The system elements have to be mobile and independent from terrestrial devices. The last prerequisite is the possibility of a dynamic reconfiguration of the network topology. In case any network node is down, it is necessary to set up new routing to provide continuous data collection and relaying. It is also assumed that a bidirectional link is built, allowing for simultaneous data gathering from the on-board instruments, as well as controlling UAVs and remotely maintaining the measurement devices supplied with sensors.

**Figure 1:**
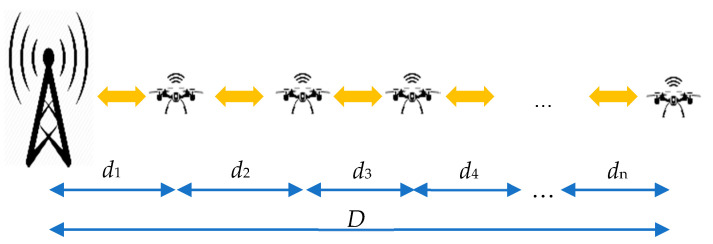
General visualization of the proposed drone sensor network; *d*_1_, *d*_2_, *d*_3_, …, *d*_n_—distances covered by subsequent drones, *D*—total length of the sensor network being the sum of distances *d*_1_, *d*_2_, *d*_3_, …, *d*_n_.

A general idea of the system is to cover the longest possible distance with network nodes carried by drones. In this section, the emphasis is on building a long network chain shown in Figure 1, in the shortest time. However, the idea of the whole project is not limited only to the line topology with theoretically endless length.

**Figure 2:**
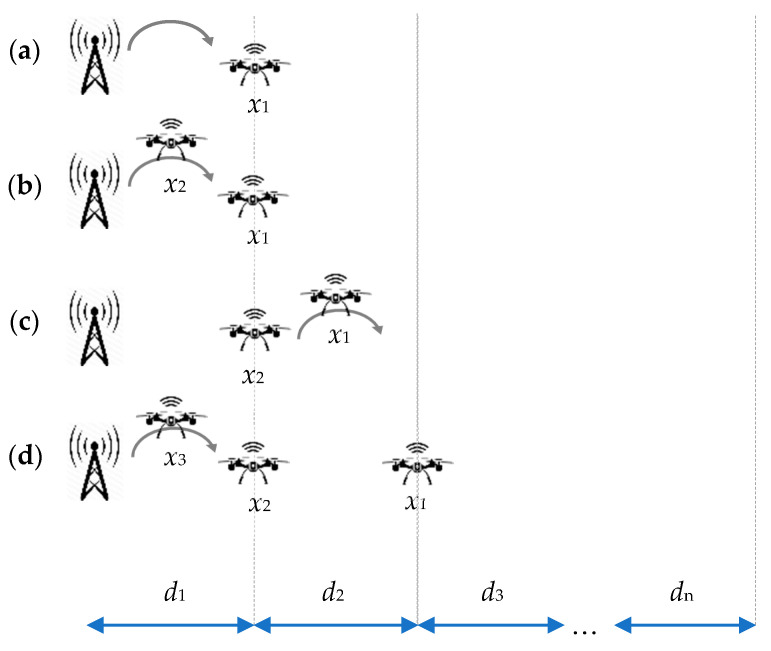
Network chain building phases: (**a**) drone *x*_1_ flies distance *d*_1;_ (**b**) drone *x*_2_ flies distance *d*_1_, drone *x*_1_ charges battery; (**c**) drone *x*_1_ flies distance *d*_2_, drone *x*_2_ charges battery; (**d**) drone *x*_3_ flies distance *d*_1_, drones *x*_1_ and *x*_2_ charge batteries, etc.

Building the network chain is performed according to the following scenario shown in Figure 2. The first drone *x*_1_ is sent from a base station. The UAV flies the longest possible distance *d*_1_ (Figure 2a). The drone ends its flight when the battery level still allows for a safe landing avoiding a cut-over in the air. After the drone has landed, the battery charging module is started. In parallel, the second drone *x*_2_ is sent from BS (Figure 2b). After charging time *t*_c_, when the battery is full and drone x_2_ has reached its destination, drone *x*_1_ repeats the flight cycle reaching distance *d*_2_ (Figure 2c). It should be emphasized here that the flight time is relatively shorter than the charging time. A complete breakdown of communication from BS to *x*_1_ or *x*_2_ during the charging process should not happen because of the usage of the redundant small accumulator or super-capacitor for radio-module powering. In case of a total blackout when the secondary battery level is low, a radio link is disabled to save energy for constant data collection from the sensors, which can be transmitted later. Collecting measurements is the highest priority task for any drone in the network. While *x*_1_ flies, *x*_2_, having a direct radio link with BS, is a network relay and provides a network signal for *x*_1_. When *x*_1_ has finished its flight, another drone x_3_ can be sent from BS and flies distance *d*_1_ (Figure 2d). When drone *x*_3_ is stationed at the distance of *d*_1_ from BS, the network chain is supported and drone x_2_ can fly another distance *d*, having communication provided by *x*_3_. The scenario shown in Figure 2 illustrates the principle of operation of the algorithm of covering distance *D*. The presented algorithm assumes only one drone in the air. In the end, the given distance *D* is divided into *n* paths *d*_1_, *d*_2_, *d*_3_, …, *d*_n_.

With all the nodes in their final positions, a radio network is set up. Now measurement data from the last node *x*_n_ is sent to BS through nodes *x*_n−1_, *x*_n−2_, …, *x*_2_, *x*_1_ respectively. If any node *x*_k_ is damaged or not responding, the network chain is broken. As a result, the data from nodes *x*_k+1_ to *x*_n_ do not reach BS. In order to bridge the gap and restore the network relaying chain, the drones have to be able to change their positions on demand. The scenario considered at this stage of the project assumes that the nodes from *x*_n_ to *x*_k+1_ are moving according to the algorithm presented in Figure 2 until the network chain is restored.

A system working under the described circumstances requires a relevant communication architecture and a routing protocol. In order to satisfy these requirements, a flying ad hoc network (FANET) with a proper architecture are considered. It is a kind of network that consists of a group of small UAVs connected in an ad-hoc manner, cooperating as a team to achieve high-level goals. The idea of FANETs confirms that all of the UAVs communicate with each other and with the BS at the same time, without having pre-defined fixed communications paths. But according to FANETs design, only a subset of UAVs can interconnect with the ground station, which perfectly satisfies the system’s idea. Following the project assumptions and the currently realized phase, a simple UAVs ad-hoc network has been chosen [23].

Considering the network architecture, a proper routing protocol has to be selected, because the system is designed to support any network topology, not only the linear one. To make it applicable, one of the six protocol groups proposed in the literature can be selected [24]:

(a) static protocols—characterized by static routing tables;
(b) proactive protocols—periodically refreshed routing tables;
(c) reactive protocols—path discovered on demand;
(d) hybrid protocols—the combination of proactive and reactive protocols;
(e) position/geographically-based protocols—based on locations or covered areas;
(f) hierarchical protocols—using the hierarchy model for routing.

The advantages of the proactive routing protocols (PRP) are that the latest information about the routes is stored and transmission delays are minimized. As far as the disadvantages are concerned, the impossibility of bandwidth optimization and a slow reaction to topology changes can be listed.

Reactive routing protocols (RRPs) are characterized by calculating a route between the nodes only if there is a connection between them. There are two types of messages in the protocol [24]: Route_Request and Route_Reply. The source node sends the Route_Request message. If any node is available in its range and receives the message, then the Route_Reply message is sent. RRP is bandwidth-efficient, but in critical cases it may take a long time to find the route.

The hybrid routing protocol (HRP) is a combination of both proactive and reactive routing protocols. Because of its flexibility, HRP seems to be the most suitable protocol for our application. RRP needs extra time to discover the route and PRP has a huge excess of control messages.

## 3. Energy Harvesting and Time of Network Chain Building

**Figure 3:**
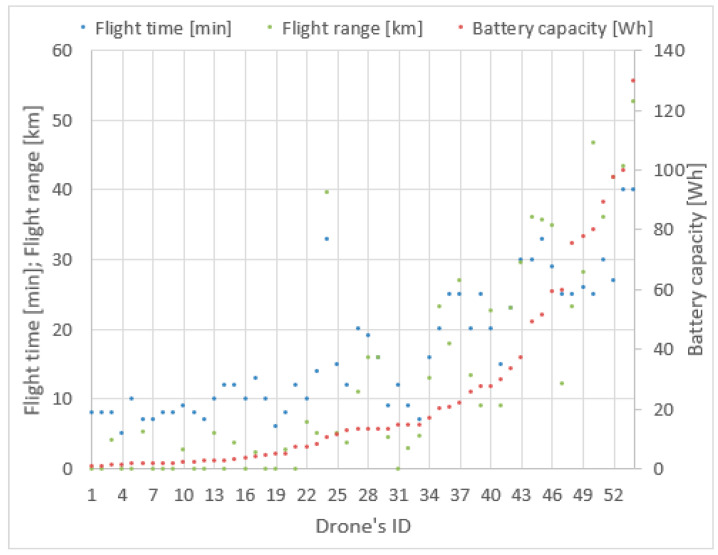
Drones parameters visualization.

Taking into consideration the advantages of multi-rotor drones, it was decided they should be used in the project. In order to find the most applicable models, an overview of popular drones on the market was prepared. The basic criteria were market availability, lifting capacity not less than 0.200 kg, the possibility of installing solar cells, sensors and additional communication equipment on board and price. Large and expensive drones with prices over 10,000 $ were not considered. Table 1 contains the specification of 54 exemplary models. The battery capacity is given in mAh, the battery voltage in Volts, the maximum flight time in minutes, the maximum flight speed in kilometers per hour and the calculated maximum flight range in kilometers. The first four parameters come from technical data sheets provided by vendors. Having flight speed *v* and flight time *t*_f_, the maximum covered distance *s* = *vt*_f_ during a single flight was assessed. The result is shown in the last column. Not all of the vendors share the flight speed in the device specifications, which resulted in gaps in Table 1. Additionally, each model has been assigned a unique order number (ID). The drones are sorted by battery capacity in ascending order. The data from Table 1 is visualized in Figure 3.

When studying Figure 3, it can be observed that the flight time and flight range vary irregularly with the increase of battery capacity given in Wh as a product of *C_b_U_b_*. Among drones with smaller battery capacity we can find models with a greater flight ranges or longer flight times. For example, the drone with ID 6 has a greater range than drones with ID 23, 26, 30, 32, 33, though the latter ones have batteries with significantly greater capacity. Thus, a simple analysis of battery capacity does not provide a clear answer as to which of the drones may be the best for the described mission purposes. The choice of a drone with a greater range also does not solve the problem, because we have to take into account the methods of energy harvesting and battery loading times.

Among known renewable energy sources, wind energy will be discussed first. The drone’s propeller can be used as an aero generator during the flight or on the ground, to provide energy to the battery [6]. As a second energy source the Sun is considered. Ji et al. described an internet of things network consisting of unmanned aerial vehicle relays [15]. The problems of limited battery life and the drone’s power consumption were solved by sharing an antenna for data transmission and energy harvesting. Transmission protocols should be designed to maximize system throughput and minimize the transmitting power. Time switching and power splitting strategies were compared in [15]. In [25] Yang et al. sought the optimal altitude of operation to find a trade-off between the covered area and harvested energy. Energy harvesting, mostly mentioned in the context of 5 G cellular networks, is applicable only in high-density energy areas [26]. Taking into account the pros and cons of the known energy sources for charging UAV batteries, it was decided to use solar cells installed on the actual drones.

The total time of building the network chain from Figure 2, called here the total journey time (TJT) can be computed by the following formula:

(1) $$TJT=\overset{n}{\underset{i=1}{\sum }}\frac{d_{i}}{v_{i}}+\left(n-1\right)t_{c},$$

where *v_i_* is the drones’ flight speeds, *n* is the number of drones used to cover distance *D*, *n* − 1 is the number of cycles of battery charges and *t*_c_ is the charging time of a single battery.

To simplify computations, identical flight time for all paths *d*_1_, *d*_2_, *d*_3_, …, *d*_n_, and identical flight speed of drones are assumed. The number of drones that cover distance *D* can be computed from formula:

(2) $$n=\lceil \frac{D}{vt_{f}}\rceil ,$$

where *v* is the flight speed of a single drone, *t_f_* is the maximum time of a single flight and ⌈ ⌉ is the ceiling function.

The effective charging time *t*_c_ is the duration of one full battery recharging cycle using a photovoltaic cell. To find the value for *t*_c_ a hypothetical endless sunshine condition was assumed. Using the above assumptions, Equation (1) reduces to:

(3) $$TJT=\frac{D}{v}+\left(n-1\right)t_{c}.$$

**Figure 4:**
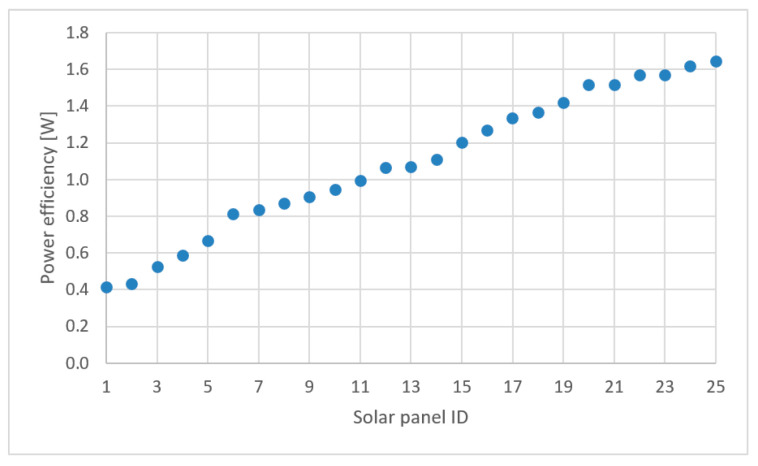
100 cm^2^ solar cell power efficiency comparison.

To assess the charging time *t*_c_, which is the critical factor for the duration of the whole mission, the parameters of the solar cells’ energy efficiency have to be known. Table 2 lists such parameters of 25 small-sized solar power cells available on the market. Each of them has different dimensions and power efficiency. Due to the different native dimensions, the shown power efficiencies are normalized to a surface area of 100 cm^2^. Table 2 is sorted by power efficiency in ascending order and illustrated in Figure 4.

According to Table 2, it can be observed that the most applicable one is the cell denoted by ID = 25, with a default surface area equal to 15.21 cm^2^. For covering the given 100 cm^2^ it is necessary to use seven panels of this size. Knowing the battery capacity *C_b_* and the nominal battery voltage *U_b_* of each drone, it is possible to estimate the charging time *t_c_* for every considered solar panel which can be computed from the formula:

(4) $$t_{c}=\frac{C_{b}U_{b}}{P_{c}},$$

where *P_c_* is the available power efficiency of the chosen solar cell.

**Figure 5:**
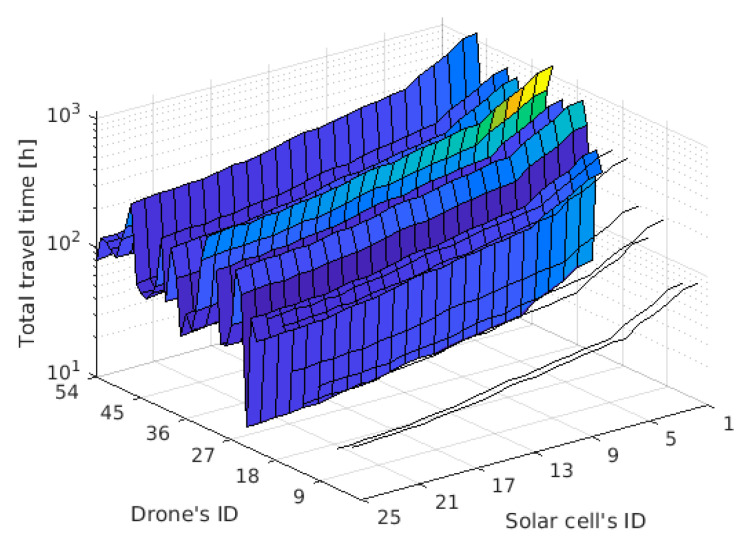
TJT while covering the distance of 100 km.

The result is a matrix of 25 (photovoltaic cells) by 54 (drones). The total journey time for the exemplary distance *D* = 100 km is shown in Figure 5.

Figure 5 contains gaps which are the result of a missing mandatory parameter—the maximum speed or flight range. The lack of this parameter reduces the list of drones from 54 to 39 units. To identify the most suitable candidates, Figure 5 was filtered and the results are presented in Table 3. It was assumed that all drones are equipped with the most efficient solar cell ID = 25 and cover the distance *D* = 100 km.

**Figure 6:**
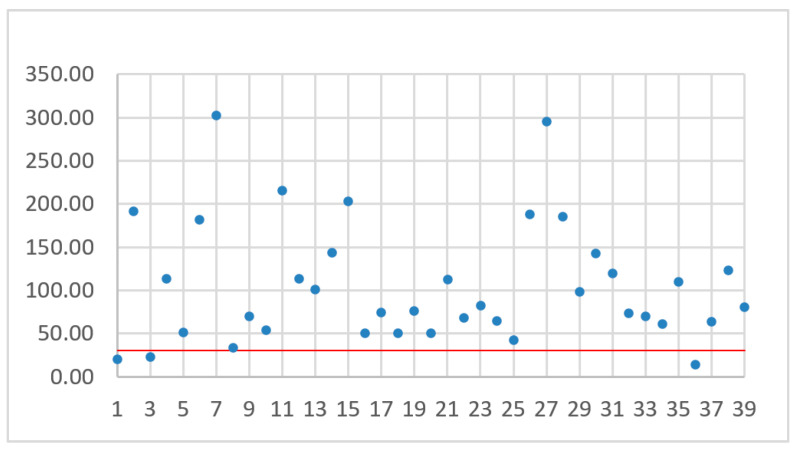
TJT with drones sorted by flight time.

**Figure 7:**
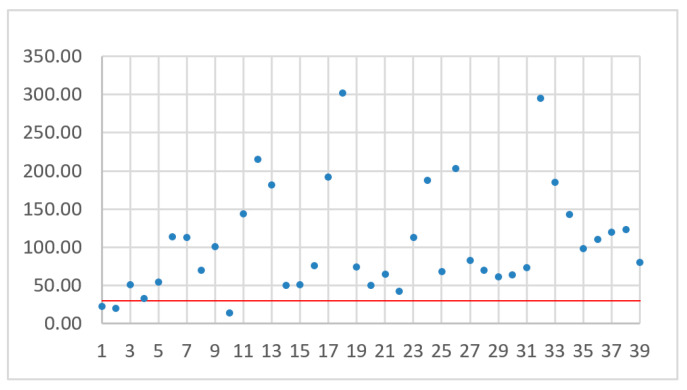
TJT with drones sorted by battery capacity.

**Figure 8:**
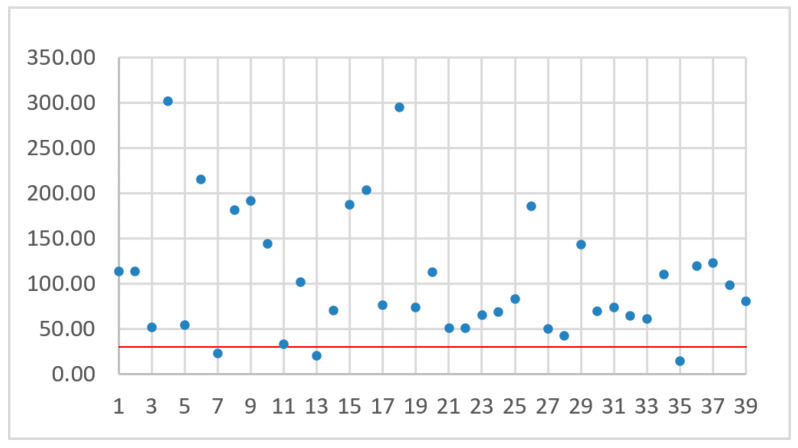
TJT with drones sorted by flight range.

**Figure 9:**
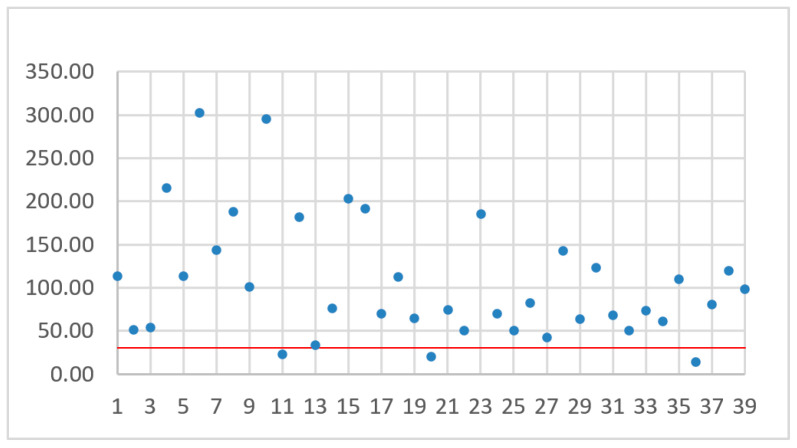
TJT with drones sorted by maximum flight speed.

The number assigned to a drone depends on the drone sorting method. The drones were sorted by flight time (Figure 6), battery capacity (Figure 7), flight range (Figure 8), and maximum flight speed (Figure 9). For all of these sorting methods TJT was computed. The goal of sorting was to find any mathematical dependence between TJT and drone parameter or parameters that can show models covering distance *d* in the shortest time.

While analyzing Figure 6, Figure 7, Figure 8 and Figure 9, no simple mathematical model nor a direct data trend can be observed. Sorting drones by the flight time, battery capacity, flight range or maximum flight speed did not give any simple dependency and the sorting criteria did not change the ranks of the best and worst drones. It can only be observed that more drones with small capacity batteries offer relatively small TJT compared with drones with higher battery capacities. On the other hand, a drone with the smallest TJT is Yuneec Mantis Q that has a relatively high battery capacity. Assuming an arbitrary limit of 30 operational hours (the red line in Figure 6, Figure 7, Figure 8 and Figure 9) for building a communication chain from Figure 2, three the most suitable and three the worst candidates were presented in Table 4 and Table 5, respectively.

## 4. Flight Range Reduction in a Function of Payload Mass

In the results presented so far the overall weight of the additional payload like measurement devices and radio relays was not taken into account. Additional payload decreases the drone’s flight speed and, as a result, the flight range is also decreased [27]. Although each of the considered drones has different characteristics like thrust, propellers diameter, weight, flight speed or battery capacity, a general dependence between flight range reduction and the drone’s payload can be derived.

To estimate flight range changes in a function of payloads mass, formulas introduced in [28,29] were used. The power consumption *P* (in kW) can be computed from equation:

(5) $$P=\frac{\left(m_{d}+m_{p}\right)v}{370\eta r}+p,$$

where $m_{d}$ is the drone’s weight in kg, $m_{p}$ the payload in kg, *v* the drone operating speed in km/h, *p* the power consumption of the onboard electronics, including sensors, in kWh, η is the power transfer efficiency for motor and propeller and r the lift to drag ratio.

The energy *E* consumed during *t* hours of flight can be described by:

(6) $$E=\frac{\left(m_{d}+m_{p}\right)vt}{370\eta r}+pt.$$

Assuming constant energy budget and small power consumption of electronics compared to total power (*p* << *P*), we obtain that:

(7) $$\left(m_{d}+m_{p}\right)v_{1}t_{1}=m_{d}v_{0}t_{0},$$

where $v_{0},t_{0}$ are the flight speed and time without load, respectively, and $v_{1},t_{1}$ are the flight speed and time with the additional payload mass. Using the dependency s=vt, we can introduce a formula that describes a flight range ratio dependent on drone and payload mass:

(8) $$\frac{s_{1}}{s_{0}}=\frac{m_{d}}{m_{d}+m_{p}},$$

where $s_{0}$ is the flight range without payload and $s_{1}$ is the flight range with a payload of mass $m_{p}$.

Equation (8) allows one to estimate a percentage of flight range decrease $R_{d}=1-\frac{s_{1}}{s_{0}}$ as a function of the payload mass to drone mass ratio $\frac{m_{p}}{m_{d}}$, i.e.:

(9) $$R_{d}=\left(1-\frac{1}{1+\frac{m_{p}}{m_{d}}}\right)100\%.$$

**Figure 10:**
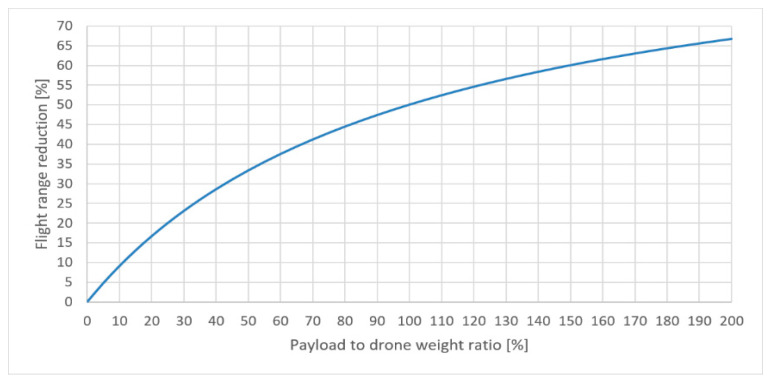
Percentage of flight range reduction as function of payload to drone’s weight ratio.

The plot of curve (9) is shown in Figure 10. The flight range is the pure flight from point A to point B, without any losses during the flight. Energy losses for reaching a cruising altitude are also omitted. The same concerns the strength and direction of the wind, or rain intensity. It is also assumed that drone flies straight away, without any obstacles on the way. The greatest value presented on horizontal axis was arbitrarily set to 200%. The precise value depends on the lifting capacity of a single drone.

The drone that offers the smallest TJT without any payload is the Yuneec Mantis Q. For a payload equal to 0.200 kg the 100 km distance is reduced to 71 km for a Yuneec Mantis Q, to 33 km for an Overmax OV-X-Bee Drone 2.4 and to 15 km for a Hubsan X4 H107D. To preserve the assumed distance of 100 km the number of drones thus has to be increased. The number of drones $n_{p}$ carrying a payload of weight $m_{p}$ can be computed from the following formula:

(10) $$n_{p}=\lceil \frac{D}{vt_{f}\left(1-R_{d}\right)}\rceil .$$

Thus, the TJT has to be recomputed for all drones, taking into account a payload mass of 0.200 kg. A new ranking of drones with the smallest and the greatest TJT for the exemplary payload mass is given in Table 6 and Table 7, respectively. Both tables were plotted repeating the assumption made by Dorling et al. [30] that when increasing payload’s weight then flight time is shortened and the drone flies with the same speed.

As it can be distinguished, the rank of the most effective and the least effective drones did not change dramatically. The Hubsan X4 H107D model was replaced by a Hubsan H501A among the drones with the shortest TJT, and the Yuneec Typhoon Q500 model was replaced by a Syma X8 PRO among the drones with the longest TJT.

From the theoretical point of view this ends our search, but it does not close the discussion on its utility. The last step is the assessment of the cost of building a drone data acquisition chain that offers the smallest TJT values. It depends on the drone’s price, number *n* of drones necessary to cover distance *D*, and the charging system cost, including the price of solar cells. If *c*_0_ is the cost of a single drone with a charging system, then the whole cost can be computed from the formula:

(11) $$c=nc_{0},$$

where *n* is computed from Equation (2). Equation (11) prefers drones with high range among drones with small TJT. Consequently, it may happen that a chain of drones that uses a model with greater but acceptable TJT costs significantly less than a chain built with a model with smaller TJT. We think that this nontechnical aspect of designing a chain of data acquisition should be an inherent element of the analysis. For example, considering the approximate cost of a chain of the length of 100 km built with drones from Table 4 or Table 6 supplied with ID 25 solar cells, the drone ranking changes. Details are presented in Table 8 and Table 9, respectively. The shown prices are only approximate because they can differ in various countries and stores. It was also assumed the unified cost of a charging system with cell ID 25 should be approximately 100$.

In both cases the winner remains the same. It is Yuneec Mantis Q. Studying Table 4, Table 6, Table 8 and Table 9 it can be observed how the payload weight changes the number of drones, and consequently, the total cost of the network chain.

The presented methodology of selecting the most suitable drone model can be summarized in the following steps:

**Input data:** The set of available drones, the set of available solar panels, the length of the network chain, the maximum mass of the payload of a single drone, and the time limit of building the chain.
   
**Step 1:** Find the most efficient solar panel.
**Step 2:** Compute the total time of building the network chain for an assumed length *D*.
**Step 3:** If the total time of building the chain exceeds the limit important for a given application, choose a new set of drones or solar panels. If not, go to the next step.
**Step 4:** Compute the total time of building the network chain with the same length for all drones carrying a payload with assumed mass.
**Step 5:** If the total time of building the chain exceeds the limit important for a given application, reduce the mass of the payload. Alternatively, we can change the set of drones or solar panels. If the computed time is less than the assumed limit, go to the next step.
**Step 6:** Compute the total cost of building the network chain for drones that guarantees the total time of building the chain less than the assumed limit.
**Step 7:** From models of drones considered in Step 6, choose a model that satisfies your technical and financial requirements.

The presented methodology allows one to identify the most suitable drone model in terms of time and the cost of building the network chain considering the total distance and the total carried payload—solar cells, radio modules and measurement instruments. The results described in this manuscript could be compared with known studies results in terms of single drone flight time carrying a payload with a given mass. Most of the research described in the literature focuses on monitoring a given area or the delivery of packages to distances not exceeding the flight range of a single drone. For example, ref. [28] introduces a formula approximating the average energy cost per kilometer while carrying a given payload mass for a given distance. The authors of [29] searched for the most optimal number of drones which minimizes the cost of package deliveries done by drones. The performance of the drone able to carry payload mass up to 0.200 kg is analyzed in [27]. The authors focused on the energy consumption considering the distance, payload mass and flight speed. Reference [31] describes the performance of three drone models while carrying a payload consisting of an onboard computer and a camera in terms of prediction of the flight time comparing regression and deep learning algorithms. Two of the drone models described in that study are also included in this manuscript.

## 5. Conclusions

Analyzing the results, no mathematical dependency between the drone parameters published by manufacturers and the TJT values was found. The sorting criteria did not change the drones’ rank estimated with the TJT parameter. When studying the details, five basic conclusions important for the applications of drones can be drawn:

- It is possible to build a network chain with a length significantly exceeding the range of any single drone in a time interval that can be considered acceptable for many applications.
- Drones can be cheap and small.
- Drones can operate in non-urbanized areas without electricity access or communication infrastructure.
- The payload mass can influence the rank of the most effective drones.
- It is impossible to choose the best models of drones studying only the values of parameters provided by manufacturers. It can result in non-optimal technical and financial decisions, which may be critical for many drone applications, e.g., during military conflicts.

The proposed solution can be applied for relaying measurement data from remote stations situated very far from the research center. The drones equipped with proper sensors and solar panels can provide information about the distribution of electromagnetic radiation, pollution, temperature, humidity, pressure, wind strength or direction, etc. in a non-urbanized area without access to the electricity grid or a communication infrastructure. Communication links built using such a drone chain, supporting rescue operations during natural disasters or military conflicts are also an advantage of the described approach. The replacement of damaged system elements is relatively easy and fast. The time required to restore the communication channels should not exceed the maximum flight time of a single drone, independently of the number of drones used, i.e., independently of distance *D*.

## Figures and Tables

**Table 1:** Considered drones.

ID	Vendor	Battery Capacity [mAh]	Battery Voltage [V]	Max. Flight Time [min]	Max. Flight Speed [km/h]	Max. Flight Range [km]	ID	Vendor	Battery Capacity [mAh]	Battery Voltage [V]	Max. Flight Time [min]	Max. Flight Speed [km/h]	Max. Flight Range [km]
1	Syma X20W	180	3.70	8	-	-	28	Overmax X-Bee Drone 8.0	1800	7.40	19	50	15.83
2	Syma X11C	200	3.70	8	-	-	29	JJRC X5	1800	7.40	16	60	16.00
3	Overmax OV-X-Bee Drone 2.4	350	3.70	8	30	4.00	30	Overmax X-Bee Drone 5.5	1800	7.40	9	30	4.50
4	Syma X21W	380	3.70	5	-	-	31	Overmax X-Bee Drone 7.2	2000	7.40	12	-	-
5	JJRC H31	400	3.70	10	-	-	32	Syma X8 PRO	2000	7.40	9	20	3.00
6	Hubsan X4 H107D	380	4.00	7	45	5.25	33	Syma X8HW	2000	7.40	7	40	4.67
7	Syma X15W	450	3.70	7	-	-	34	DJI Spark	1480	11.40	16	49	13.07
8	Syma X5SW Explorers 2	500	3.70	8	-	-	35	Hubsan H501A	2700	7.40	20	70	23.33
9	Syma X23W	500	3.70	8	-	-	36	Parrot Anafi	2700	7.60	25	43	17.92
10	Parrot Mambo fly	550	3.70	9	18	2.70	37	XIAOMI FIMI A3	2000	11.10	25	65	27.08
11	Syma X5HW	600	3.70	8	-	-	38	JJRC X11	3400	7.60	20	40	13.33
12	Syma X54HW	650	3.70	7	-	-	39	JJRC X12	2400	11.40	25	21.6	9.00
13	Parrot Mambo Mission	660	3.70	10	30	5.00	40	DJI Mavic Air	2375	11.55	20	68	22.67
14	Overmax X-Bee Drone 3.1	750	3.70	12	-	-	41	Cheerson CX-20	2700	11.10	15	36	9.00
15	uGo Sirocco	800	3.70	12	18	3.60	42	Hubsan H117S Zino	3000	11.10	23	60	23.00
16	XIAOMI MI DRONE MINI	920	3.80	10	-	-	43	Parrot BEBOP 2 POWER	3350	11.10	30	59	29.50
17	DJI Ryze Tello	1100	3.80	13	11	2.38	44	Autel EVO	4300	11.40	30	72	36.00
18	Overmax X-Bee Drone 6.1	600	7.40	10	-	-	45	XIAOMI FIMI X8 SE	4500	11.40	33	65	35.75
19	Xblitz DISCOVER	650	7.40	6	-	-	46	DJI Mavic 2 Pro	3850	15.40	29	72	34.80
20	TKKJ TK116W	1300	3.70	8	20	2.67	47	Yuneec Typhoon Q500	5400	11.10	25	29	12.08
21	Syma X25 PRO	1000	7.40	12	-	-	48	GoPro Karma	5100	14.80	25	56	23.33
22	Goclever Drone Predator FPV	2000	3.70	10	40	6.67	49	XIAOMI Mi Drone 4K	5100	15.20	26	65	28.17
23	JJRC H73	1100	7.60	14	22	5.13	50	Yuneec Typhoon H	5400	14.80	25	112	46.67
24	Yuneec Mantis Q	2800	3.70	33	72	39.60	51	DJI Phantom 4 Pro	5870	15.20	30	72	36.00
25	JJRC X9	1000	11.40	15	20	5.00	52	DJI Inspire 2	4280	22.80	27	93	41.85
26	Yuneec Breeze	1150	11.10	12	18	3.60	53	DJI Matrice 600	4500	22.20	40	65	43.33
27	Overmax X-Bee Drone 9.0 GPS	1800	7.40	20	33	11.00	54	DJI Matrice 100	5700	22.80	40	79	52.67

**Table 2:** Considered solar cells.

Index	Dimensions [mm]	Power Efficiency [W/100 cm^2^]	Index	Dimensions [mm]	Power Efficiency [W/100 cm^2^]
1	120 × 60 × 0.8	0.42	14	95 × 95	1.11
2	154 × 45	0.43	15	50 × 50	1.2
3	53 × 18 × 2.5	0.52	16	125 × 63	1.27
4	112 × 91 × 3	0.59	17	255 × 147 × 2	1.33
5	136 × 110 × 3	0.67	18	80 × 55	1.36
6	255 × 145 × 9	0.81	19	65 × 65	1.42
7	60 × 60	0.83	20	110 × 60 × 2.5	1.52
8	20 × 23	0.87	21	120 × 110 × 2	1.52
9	115 × 115 × 3	0.91	22	165 × 135 × 3	1.57
10	53 × 30	0.94	23	165 × 135 × 3	1.57
11	65 × 65 × 3	0.99	24	52 × 19	1.62
12	30 × 25	1.07	25	39 × 39	1.64
13	100 × 28	1.07			

**Table 3:** Drones combined with the most effective solar cell (ID = 25).

Drone	Max. Flight Time [min]	Battery Capacity *C_b_U_b_* [Wh]	Max. Flight Range [km]	Max. Flight Speed [km/h]	No. by TJT	No. by Flight Time	No. by Batt. Capacity	No. by Flight Range	No by Max Flight Speed	Total Journey Time [h]
Yuneec Mantis Q	33.00	10.36	39.60	72.00	1	36	10	35	36	13.99
Hubsan X4 H107D	7.00	1.52	5.25	45.00	2	1	2	13	20	19.79
Overmax OV-X-Bee Drone 2.4	8.00	1.30	4.00	30.00	3	3	1	7	11	23.03
Parrot Mambo Mission	10.00	2.44	5.00	30.00	4	8	4	11	13	33.05
XIAOMI FIMI A3	25.00	22.20	27.08	65.00	5	25	22	28	27	42.06
Hubsan H501A	20.00	19.98	23.33	70.00	6	20	20	27	32	50.05
JJRC X5	16.00	13.32	16.00	60.00	7	16	14	22	25	50.29
Overmax X-Bee Drone 8.0	19.00	13.32	15.83	50.00	8	18	15	21	22	50.62
Parrot Mambo fly	9.00	2.04	2.70	18.00	9	5	3	3	2	51.37
uGo Sirocco	12.00	2.96	3.60	18.00	10	10	5	5	3	54.18
Autel EVO	30.00	49.02	36.00	72.00	11	34	29	33	34	61.04
XIAOMI FIMI X8 SE	33.00	51.30	35.75	65.00	12	37	30	32	29	63.96
Parrot Anafi	25.00	20.52	17.92	43.00	13	24	21	23	19	64.75
DJI Mavic Air	20.00	27.43	22.67	68.00	14	22	25	24	31	68.23
Parrot BEBOP 2 POWER	30.00	37.19	29.50	59.00	15	33	28	30	24	69.56
Goclever Drone Predator FPV PRO	10.00	7.40	6.67	40.00	16	9	8	14	17	70.03
DJI Mavic 2 Pro	29.00	59.29	34.80	72.00	17	32	31	31	33	73.53
DJI Spark	16.00	16.87	13.07	49.00	18	17	19	19	21	73.90
Overmax X-Bee Drone 9.0 GPS	20.00	13.32	11.00	33.00	19	19	16	17	14	75.97
DJI Matrice 100	40.00	129.96	52.67	79.00	20	39	39	39	37	80.33
Hubsan H117S Zino	23.00	33.30	23.00	60.00	21	23	27	25	26	82.71
Yuneec Typhoon H	25.00	79.92	46.67	112.00	22	29	35	38	39	98.14
JJRC H73	14.00	8.36	5.13	22.00	23	13	9	12	9	101.18
DJI Phantom 4 Pro	30.00	89.22	36.00	72.00	24	35	36	34	35	109.96
JJRC X11	20.00	25.84	13.33	40.00	25	21	23	20	18	112.55
TKKJ TK116W	8.00	4.81	2.67	20.00	26	4	7	2	5	113.28
DJI Ryze Tello	13.00	4.18	2.38	11.00	27	12	6	1	1	113.36
DJI Inspire 2	27.00	97.58	41.85	93.00	28	31	37	36	38	119.82
DJI Matrice 600	40.00	99.90	43.33	65.00	29	38	38	37	30	123.10
XIAOMI Mi Drone 4K	26.00	77.52	28.17	65.00	30	30	34	29	28	143.03
JJRC X9	15.00	11.40	5.00	20.00	31	14	11	10	7	143.72
Overmax X-Bee Drone 5.5	9.00	13.32	4.50	30.00	32	6	13	8	12	181.62
GoPro Karma	25.00	75.48	23.33	56.00	33	28	33	26	23	185.47
JJRC X12	25.00	27.36	9.00	21.60	34	26	24	15	8	187.73
Syma X8HW	7.00	14.80	4.67	40.00	35	2	17	9	16	191.59
Cheerson CX-20	15.00	29.97	9.00	36.00	36	15	26	16	15	203.35
Yuneec Breeze	12.00	12.77	3.60	18.00	37	11	12	6	4	215.24
Yuneec Typhoon Q500	25.00	59.94	12.08	29.00	38	27	32	18	10	295.19
Syma X8 PRO	9.00	14.80	3.00	20.00	39	7	18	4	6	302.14

**Table 4:** The most effective drones combined with the most efficient solar cell (ID = 25).

Rank	Model	Total Journey Time [h]	Max. Flight Time [min]	Battery Capacity [Wh]	Max. Flight Range [km]	Max. Flight Speed [km/h]	Weight [kg]
1	Yuneec Mantis Q	13.99	33.00	10.36	39.60	72.00	0.480
2	Hubsan X4 H107D	19.79	7.00	1.52	5.25	45.00	0.035
3	Overmax OV-X-Bee Drone 2.4	23.03	8.00	1.30	4.00	30.00	0.100

**Table 5:** The least effective drones combined with the most efficient solar cell (ID = 25).

Rank	Model	Total Journey Time [h]	Max. Flight Time [min]	Battery Capacity [Wh]	Max. Flight Range [km]	Max. Flight Speed [km/h]	Weight [kg]
39	Syma X8 PRO	302.14	9.00	14.80	3.00	20.00	0.760
38	Yuneec Typhoon Q500	295.19	25.00	59.94	12.08	29.00	1.700
37	Yuneec Breeze	215.24	12.00	12.77	3.60	18.00	0.385

**Table 6:** The most effective drones combined with the most efficient solar cell (ID = 25), with payload of 0.200 kg.

Rank	Model	Total Journey Time [h]	Max. Flight Time [min]	Battery Capacity [Wh]	Max. Flight Range [km]	Max. Flight Speed [km/h]	Weight [kg]
1	Yuneec Mantis Q	20.88	23.00	10.36	27.95	72.00	0.680
2	Hubsan H501A	62.78	14.00	19.98	16.67	45.00	0.700
3	Overmax X-Bee Drone 8.0	67.60	14.00	13.32	11.44	30.00	0.720

**Table 7:** The least effective drones combined with the most efficient solar cell (ID = 25) with payload of 0.200 kg.

Rank	Model	Total Journey Time [h]	Max. Flight Time [min]	Battery Capacity [Wh]	Max. Flight Range [km]	Max. Flight Speed [km/h]	Weight [kg]
39	DJI Ryze Tello	403.11	4.00	14.80	0.68	11.00	0.280
38	Syma X8 PRO	384.50	7.00	59.94	2.38	20.00	0.960
37	Yuneec Breeze	334.62	8.00	12.77	2.37	18.00	0.585

**Table 8:** The approximate cost of a chain of drones supplied with a charging system with solar cells ID 25, *D* = 100 km, without additional payload.

Rank	Model	*n*	*c*_0_ [$]	*c* [$]
1	Yuneec Mantis Q	3	≈600	1800
2	Overmax OV-X-Bee Drone 2.4	25	≈150	3700
3	Hubsan X4 H107D	20	≈250	5000

**Table 9:** The approximate cost of a chain of drones supplied with a charging system with solar cells ID 25, *D* = 100 km, payload mass equal to 0.200 kg.

Rank	Model	*n_p_*	*c*_0_ [$]	*c_p_* [$]
1	Yuneec Mantis Q	4	≈600	2400
2	Hubsan H501A	6	≈400	2400
3	Overmax X-Bee Drone 8.0	9	≈300	2700

## Data Availability

Data available in a publicly accessible repository that does not issue DOIs.
